# The Urine Calcium/Creatinine Ratio and Uricemia during Hyponatremia of Different Origins: Clinical Implications

**DOI:** 10.3390/jcm12020723

**Published:** 2023-01-16

**Authors:** Guy Decaux, Wim Musch

**Affiliations:** 1 Research Unit for the Study Hydromineral Metabolism, Erasmus University Hospital, 1070 Brussels, Belgium; 2 Department of Internal Medicine, Molière-Longchamps Hospital, 1190 Brussels, Belgium; 3 Department of Internal Medicine, Bracops Hospital, 1070 Brussels, Belgium

**Keywords:** hyponatremia, calciuria, uricemia, SIADH, polydipsia, thiazides

## Abstract

Background: Chronic hyponatremia is known to be associated with osteoporosis. It has been shown that chronic hyponatremia increases bone resorption in an attempt to release body stores of exchangeable sodium by different mechanisms. We wanted to know the calciuria of patients with hyponatremia of different origins. Material and Methods: We made a retrospective study of 114 consecutive patients with asymptomatic hyponatremia of different origins with the usual serum and urine chemistry. Result: In hyponatremia due to SIADH, we had a high urine calcium/creatinine ratio of 0.23 ± 0.096 while in patients with salt depletion the UCa/UCr ratio was low (0.056 ± 0.038), in patients with hyponatremia secondary to thiazide intake the value was also low (0.075 ± 0.047) as in hypervolemic patients (0.034 ± 0.01). In hyponatremia due to polydipsia, the value was high (0.205 ± 0.10). Correction of hyponatremia in the euvolemic patients was associated with a significant decrease in the UCa/UCr ratio. In patients with hyponatremia secondary to thiazide intake, we noted that in the patients with low uric acid levels (<4 mg/dL, suggesting euvolemia) we also observed a low UCa/UCr (<0.10). In nine patients with chronic SIADH (SNa 125.1 ± 3.6 mEq/L), the 24 h urine calcium excretion was 275 ± 112 mg and decreased to 122 ± 77 mg (*p* < 0.01) after at least 2 weeks of treatment. Conclusions: Patients with chronic hyponatremia due to SIADH usually have a high UCa/UCr ratio (>0.15). This is also observed in hyponatremia secondary to polydipsia. Patients with thiazide-induced hyponatremia usually have low UCa/UCr levels and this is the case even among those with a biochemistry similar to that in SIADH (uric acid < 4 mg/dL).

## 1. Introduction

Chronic hyponatremia is well known to be associated with osteoporosis and bone fractures [1]. This osteoporosis was first shown in rats [2]. Many factors contribute to this complication. A direct effect of low SNa on in vitro bone composition has been shown as a result of the activation of the osteoclast [3]. Chronic hyponatremia is usually associated with an increase in ADH and this could also stimulate bone resorption as receptors both for V1 and V2 have been described on the osteoclast [4]. The two AVPr receptors, AVPr 1α and AVPr 2, are expressed in osteoblasts and osteoclasts and they enhanced and reduced, respectively, the formation of bone-resorbing osteoclasts and bone-forming osteoblasts [4]. Chronic hyponatremia is also associated with sarcopenia [5,6,7], which contributes to osteoporosis [8]. In idiopathic hypercalciuria, osteoporosis is also reported [9] and in patients with central diabetes insipidus a hypercalciuria is also observed (and attributed to the high diuresis) [10].

High calciuria has been reported in hyponatremia secondary to the syndrome of inappropriate secretion of antidiuretic hormone (SIADH) [11,12]. This has been attributed to the increase in the effective vascular volume [11,12]. This high calciuria could contribute to osteoporosis. A similar observation has been reported in primary aldosteronism, and also assigned to the increase in effective volemia [13,14,15]. We know that tubular resorption of calcium is associated with sodium resorption [16]. In SIADH, the increase in effective volemia will decrease the tubular resorption of sodium at different levels of the renal tubules and this will contribute to the increase in calciuria (particularly in the proximal tubule [17,18]). In a previous study on hypercalciuria in SIADH, there were only two patients reported where daily calcium excretion was reported after correction of SNa [11] and one in another study [12]. In the present report, we discuss the calciuria of a large population of patients with chronic hyponatremia of different origins. We also compare the urine calcium/creatinine (UCa/UCr) ratio obtained in a spot urine sample with uricemia in the diagnosis of hyponatremia of different origins.

## 2. Materials and Methods

This is an observational retrospective study which was performed in two units of 30 beds of our departments of internal medicine. We analyzed, before any treatment, over the five last years (2016–2020), all the patients admitted with asymptomatic hyponatremia. We included only patients with SNa ≤ 132 mEq/L. Patients with a history of fall were included if there were no other symptoms secondary to hyponatremia mentioned in the dossier. We included only patients where we had measured, in a morning serum and urine sample (before breakfast), the osmolality, the serum Na^+^, K^+^, Cl^−^, CO_2_T, the total protein concentration and the concentrations of urea, creatinine, uric acid and calcium and phosphorus. The most common reason for exclusion of a patient was the lack of measurement of urine calcium during the period of hyponatremia.

We divided the patients into five groups: the euvolemic patients (SIADH), the salt depletion patients not due to diuretic intake (SD), the patients in whom hyponatremia was due to diuretic intake (all thiazides), the polydipsic patients whether due to water intake or due to beer potomania. For the polydipsic patients, we included only those with a history of a fluid intake greater than 4 L/day and with a urine osmolality lower than 200 mOsm/kg H_2_O. The last group was the hypervolemia secondary to cirrhosis or cardiac failure patients. Patients who took oral calcium were not included in the study. All measurements were performed by classical methods in our laboratory, but free serum calcium was unfortunately not available.

Statistical analysis was performed with GraphPad software. A Kolmogorov–Smirnov test was first applied to determine if the analyzed variables were normally distributed. Comparisons between means were performed with Student’s *t*-test or paired Student’s *t*-test when appropriate. The χ^2^ test was also used. A *p* value < 0.05 was considered as statistically significant. Data are presented as mean ± SD. This retrospective study was approved by the Ethics Committee of Hospital Erasme (Ref. P2020/062).

## 3. Results

**Figure 1:**
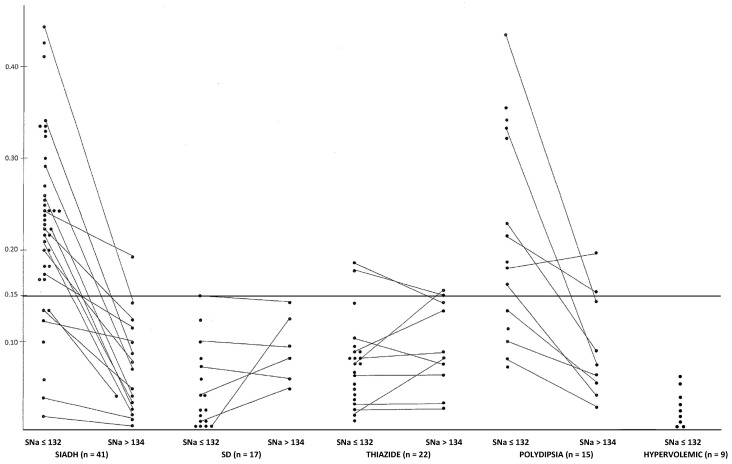
Evolution of the UCa/UCr ratio before and after correction of hyponatremia of different origins (the horizontal line represents the upper normal limit obtained in adults before breakfast).

Figure 1 illustrates that only patients with SIADH or polydipsia frequently presented higher values of UCa/UCr ratio during hyponatremia.

### 3.1. SIADH Patients

Table 1 shows the mean values of each class of hyponatremic patients where we had a urine UCa/UCr measurement in at least six patients. In our 41 patients with SIADH (15 women and 26 men), it was due to cancer (mainly pulmonary; values only retained for patients without bone metastasis and before chemotherapy), due to medications (mainly carbamazepine and SSRI), due to chronic pulmonary disease, due to brain damage (mostly tumors) and idiopathic. The mean age was 61 ± 15 years and the mean value of UCa/UCr was 0.23 ± 0.096. It should be noted than the average uricemia of women with hyponatremia due to SIADH was lower than in men (2.4 ± 0.7 mg/dL and 2.9 ± 0.8 mg/dL; *p* < 0.05). On the other hand, the UCa/UCr ratio was the same for women (0.24 ± 0.10) and men (0.22 ± 0.09). Of these 41 patients with SIADH, we had four patients (all men) with a uric acid level higher than 4 mg/dL (10%). Twenty-nine of the 41 patients had a UCa/UCr ratio ≥ 0.20 (70%), 34 of the 41 patients had a value > 0.15 (83%) and 37 of the 41 patients had a value ≥ 0.11 (90%) (see Figure 1). No correlation was observed between SNa and UCa/UCr (r = 0.13; NS) or between FE.Na and UCa/UCr (r = 0.04). Among the 41 patients, six patients had low natriuria (FE.Na 0.07 ± 0.04% and UNa 28 ± 17 mEq/L). All six patients had high calciuria (UCa/UCr 0.27 ± 0.07). The mean serum calcium value of these 41 patients was 9.0 ± 0.55 mg/dL and total protein concentration was 6.6 ± 0.5 g/dL (concomitant albumin concentration was unfortunately not available in most patients). Patients with SIADH also frequently had low serum urea levels as expected. In 15 of these patients, in whom it was available, we observed a significant decrease in morning UCa/UCr ratio value with the normalization of SNa (see Table 2: initial value of UCa/UCr 0.25 ± 0.1 and after normalization of SNa it was reduced to 0.09 ± 0.05 (*p* < 0.001)). In five patients with mild hyponatremia (SNa ≥ 130–≤132 mEq/L), we also observed an abnormally high UCa/UCr ratio (0.26 ± 0.06). Nine patients had a 24 h urine collection before and after SNa correction (Table 3) and we observed a decrease in urine calcium excretion from 275 ± 112 mg/24 h to 122 ± 77 mg/24 h (*p* < 0.01) (Table 3).

### 3.2. Salt Depletion

In the 17 patients (9 were women) with hyponatremia due to salt depletion (all due to digestive solute lost), we observed a low UCa/UCr value (see Figure 1 and Table 1 and Table 2). As expected, these patients presented higher urea and uric acid levels than in the SIADH patients. Mean urine sodium concentrations were also much lower.

### 3.3. Diuretics

In patients with hyponatremia secondary to thiazide intake (13 were women), we observed much lower UCa/UCr ratios than in patients with SIADH (see Table 1, Table 2 and Table 3 and Figure 1).

In five patients under chronic furosemide therapy (three cirrhosis and two cardiac failure), we had a UCa/UCr ratio 2–4 h after the intake and we observed in these five patients a UCa/UCr value ≥ 0.28, while in four patients (two cirrhosis and two cardiac failure) in whom we had a measurement just before furosemide intake, all had a UCa/UCr ratio < 0.10. These patients were not included in the tables.

Patients under thiazide treatment (*n* = 22) had higher calcium and protein concentrations compared with SIADH patients (see Table 1). The normalization of SNa in these patients (Table 2) was associated with a decrease in calcium (from 9.45 ± 0.6 mg/dL to 9.0 ± 0.25 (*p* < 0.05) and a decrease in total serum protein from 7.6 ± 0.5 to 6.4 ± 0.4 g/dL (*p* < 0.001) (this was carried out by isotonic saline administration). All these patients were asymptomatic.

We had eight patients with a serum uric acid level lower than 4 mg/dL, which could suggest SIADH if we did not know that these patients were taking a thiazide. Only one of these eight patients presented a UCa/UCr ratio of 0.18, however all seven others presented a low UCa/UCr ratio (<0.10) (see Figure 1). In the SIADH group, only 3 of the 41 patients presented a UCa/UCr ratio lower than 0.10, which was observed in 18 of the 22 patients on thiazides (*p* < 0.001) (see Figure 1).

It is well known that in SIADH, correction of hyponatremia is associated with an increase in serum uric acid level while in thiazide-induced hyponatremia correction of SNa is associated with a decrease in serum urate (see Table 2) and this occurs even in those with a low uric acid level (the eight patients decreased their uric acid further with the correction of SNa). In the patients where hyponatremia was due to thiazide intake, seven patients presented a low K concentration (<3.5 mEq/L).

### 3.4. Polydipsia

The mean UCa/UCr ratio in the polydipsic patients was high at 0.205 ± 0.10 (nine women) (Table 1) and in the nine patients we had the value of UCa/UCr after normalization of SNa (Table 2). Eight of the nine patients decreased their UCa/UCr value (see Figure 1). The lowest urea values were observed in the polydipsic patients (Table 1).

In six polydipsic patients, we observed a serum uric acid level higher than 5 mg/dL. All these patients were “beer drinkers” (and also had high UCa/UCr ratio). All the compulsive water drinkers presented a uric acid level lower than 4 mg/dL. Four patients presented a low potassium level (<3.5 mEq/L).

### 3.5. Hypervolemic Hyponatremia due to Cirrhosis (n = 7) or Cardiac Failure (n = 2) (see Table 1 and Figure 1)

The values of UCa/UCr observed were very low in all these patients (mean value 0.034 ± 0.01). All these patients were on spironolactone therapy (four patients were women).

## 4. Discussion

Hyponatremia secondary to SIADH is the most frequent cause of chronic hyponatremia. The effective volemia in SIADH is increased which will induce a decrease in renal tubular resorption of sodium. The tubular urate reabsorption, coupled with the sodium reabsorption, is mainly localized in the proximal tubule, where the decrease in sodium reabsorption will explain the high urate clearance and the low uricemia typically reported [17,18,20]. The first observation of a low uricemia in a SIADH patient was reported by Laurence Beck more than 40 years ago [17]. He noted that 90% of the patients with SIADH presented an uricemia level lower than 4 mg/dL. In this previous report, patients with hyponatremia secondary to diuretics (mainly thiazides) were not included. In a previous publication on 110 consecutive patients with hyponatremia, 33 were due to SIADH [20]. Seven of these 33 patients (28%) presented a uric acid level higher than 4 mg/dL [20]. In this previous study, the mean age was 70 ± 14 years. We know that uric acid increases with age, particularly in women [21]. In the present study, the mean age of our 41 patients with SIADH was younger (61 ± 15 years). We observed only four patients with a uric acid level higher than 4 mg/dL (10%), and three of these four patients presented a UCa/UCr ratio higher than 0.17 (see later). In the present study of 114 patients with hyponatremia (the 5 patients on furosemide excluded), we had 22 patients where hyponatremia was due to thiazide intake. Eight of these twenty-two patients presented a uric acid level lower than 4 mg/dL (like in SIADH) and seven of these eight patients presented a low UCa/UCr ratio (<0.10) which is unusual in SIADH (three of the forty-one patients) (see Figure 1). Another difference between the SIADH patients and the patients with thiazide-induced hyponatremia is the evolution of serum uric acid: in SIADH, uric acid increases with the correction of SNa, while in thiazide-related hyponatremia, the serum uric acid decreases with the correction of SNa, even in the eight patients with low uric acid levels. It has been shown that even in patients with hyponatremia secondary to thiazide intake and presenting a biology of SIADH, the solute deficit is the main factor explaining the decrease in serum sodium [22] and not water retention like in SIADH [23].

The data show that another factor than uric acid excretion is highly sensitive to variation in the “effective volemia”, namely the calcium excretion. All our patients with hyponatremia due to SIADH decreased their UCa/UCr ratio with the correction of SNa (see Figure 1). This was also observed in the patients with hyponatremia secondary to polydipsia (except in 1 of the 15 patients, see Figure 1). We know that more than 95% of the filtered calcium is reabsorbed along the renal tubules. In the proximal tubules, 60–70% of filtered calcium is reabsorbed by passive mechanisms. In the thick ascending limb, 29% of calcium is reabsorbed by paracellular diffusion. The fine regulation of calcium excretion occurs in the distal convoluted tubules and connecting tubules despite the fact that only 10–15% of filtered calcium is reabsorbed here [16]. Expansion of the extracellular fluid is associated with an increase in sodium, chloride and calcium excretion, whereas reciprocal effects are seen with volume contraction. In our patients with SIADH treated with urea at relatively small doses (15 to 30 g/day), the decrease in calciuria is likely due to the normalization of the effective volemia while high doses of urea administered in normal subjects induce an increase in calciuria (osmotic diuresis) [24]. Interestingly hypercalciuria was recently reported in patients with central diabetes insipidus (CDI) when untreated [10]. This underscores that urine flow rates and urinary Na handling might be more important than a direct effect of AVP. This could also explain why hypercalciuria is observed in polydipsic schizophrenic patients [25]. In CDI, an increase in serum uric acid level is usually observed (and attributed to a lack of V1 effect) while in primary polydipsia the serum uric acid level is usually low [26].

We had the urine UCa/UCr ratios of five patients in the first hours after furosemide intake and they confirmed very high levels (≥0.28). It is well known that chronic furosemide intake is associated with chronic hypercalciuria which contributes to osteoporosis [27]. Hyponatremia secondary to SIADH produces chronic hypercalciuria. The cause of the additional urinary calcium loss must be either of bone mineral and/or intestinal origin. In rats with chronic hypercalciuria induced by chronic furosemide intake, the intestinal calcium absorption increased enough to allow a high urine calcium excretion without affecting bone composition [27].

It must be recalled that during pregnancy and lactation the enhanced intestinal calcium absorption serves to provide calcium for fetal development and lactogenesis and this is due to the hormone prolactin [28].

Even in patients with mild hyponatremia (mean SNa 131 mEq/L), we observed a high calciuria. Treatment of mild hyponatremia will likely contribute to bone recovery.

In a single study on hyponatremia secondary to SIADH, the levels of 1–250 H vitamin D and of PTH were reported to be normal [12].

The serum calcium was higher in our patients with hyponatremia secondary to thiazides than in the patients with SIADH. This could be explained by a higher protein concentration in patients on thiazides (due to the low effective volemia). Even in patients with cirrhosis or cardiac failure, we measured both very low calciuria and low serum calcium levels, which was due to the low albumin concentration in these patients.

A recent article has reported that chronic hyponatremia (mainly due to SIADH) is associated with a higher risk of kidney stones [29].

A limitation of our study is its retrospective nature. A prospective study should be carried out with a better selection of patients who are symptomatic or not. A study should compare if the UCa/UCr determination is more sensitive than the uricemia (or FE uric acid) in detecting hyponatremia due to SIADH. A prospective study should also be carried out to see if corrections of mild hyponatremia allow a decrease in osteoporosis.

In patients with SIADH or polydipsia, moderate or mild hyponatremia is associated with hypercalciuria. Further study is warranted to see if excess urinary calcium comes primarily from bone and/or the digestive tract. It is not excluded that, in our patients with chronic hypervolemic hyponatremia or chronic hyponatremia related to thiazide intake (mainly thiazides or spironolactone), there is a decrease in the digestive absorption of calcium to avoid hypercalcemia. Further study needs to be carried out.

## Figures and Tables

**Table 1:** Some morning biochemical data in patients with hyponatremia secondary to syndrome of inappropriate secretion of antidiuretic hormone (SIADH), to salt depletion (SD), to thiazides, to polydipsia and to hypervolemia (cirrhosis/cardiac failure).

Controls in Adults	SIADH (*n* = 41)	SD (*n* = 17)	Thiazides (*n* = 22)	Polydipsia (*n* = 15)	Cirrhosis (7) Cardiac Failure (2) (*n* = 9)
SNa (mEq/L) (135–145)	123.3 ± 4.5	125.2 ± 3.3	123.4 ± 4.5	123.2 ± 4.4	127 ± 2.3
Urea (mg/dL) (17–42)	22.1 ± 8.4	35 ± 18 *	42 ± 18 *	20.4 ± 10 **	28 ± 19
Uric acid (mg/dL) (2.5–7)	2.7 ± 0.9	5.4 ± 1.9 *	4.8 ± 1.85 *	4.6 ± 2.25	4.7 ± 2.2
Uosm (mOsm/kgH_2_O) (50–1100)	462 ± 123	552 ± 155	432 ± 132	122 ± 40	450 ± 140
UNa (mEq/L) (<170)	89 ± 39	24 ± 14 *	45 ± 24 *	13 ± 14 *	25 ± 20 *
SProt (g/dL) (6–8)	6.6 ± 0.5	7.0 ± 0.8	7.4 ± 0.6 *	7.0 ± 0.8	6.5 ± 1.0
SCa (mg/dL) (8.1–10.6)	9.0 ± 0.55	9.03 ± 0.50	9.40 ± 0.64 ***	9.12 ± 0.66	8.3 ± 0.8
UCa/UCr (before breakfast: ≤0.15)	0.23 ± 0.096	0.056 ± 0.038 *	0.075 ± 0.047 *	0.205 ± 0.10 **	0.034 ± 0.01 *

Note: 1 mg/dL of calcium = 0.25 mmol/L and 1 mg/dL of creatinine = 0.01131 µmol/L. * *p* < 0.001 compared to SIADH; ** *p* < 0.01 compared to SD and thiazide; *** *p* < 0.01 compared to SIADH.

**Table 2:** Evolution of UCa/UCr ratio before and after correction of SNa (mean ± SD).

	SIADH (*n* = 15)		SD (*n* = 6)		Thiazides (*n* = 10)		Polydipsia (*n* = 9)	
SNa (mEq/L)	124 ±3.6	137 ± 2.2 *	126 ± 2.4	134 ± 1.3 *	123 ± 4.9	134 ± 1.6 *	121.5 ± 5	136 ± 3 *
SUrea (mg/dL)	24 ± 11	39 ± 13 *	36 ± 8	25 ± 6.7 ***	39.3 ± 18	33 ± 10	19 ± 9	17 ± 7
SUric acid (mg/dL)	3 ± 0.45	4 ± 1.37 *	5.7 ± 2	4.6 ± 1.5 ***	5 ± 2.1	4.1 ± 2 *	4.3 ± 2.4	4.5 ± 1.5
Uosm (mOsm/kgH_2_O)	391 ± 87	703 ± 240 *	650 ± 135	604 ± 180	392 ± 120	430 ± 170	125 ± 40	306 ± 150 **
SCa (mg/dL)	9.15 ± 0.33	9.44 ± 0.40	9.16 ± 0.52	8.7 ± 0.17	9.45 ± 0.6	9.0 ± 0.25 ***	9.0 ± 0.5	8.8 ± 0.5
SPO_4_ (mg/dL)	3.5 ± 0.5	3.8 ± 0.8	3.2 ± 0.9	3.1 ± 0.5	3.2 ± 0.6	2.9 ± 0.6	3.3 ± 1	3.2 ± 0.6
UCa/UCr	0.25 ± 0.1	0.09 ± 0.05 *	0.06 ± 0.03	0.064 ± 0.036	0.09 ± 0.05	0.07 ± 0.05	0.21 ± 0.10	0.10 ± 0.07 ***

* *p* < 0.001; ** *p* < 0.01; *** *p* < 0.05.

**Table 3:** Evolution of 24 h urine calcium excretion before and after normalization of serum sodium (SNa) in 9 patients with SIADH (data obtained after at least 2 weeks of treatment).

Sex—Age (Year)—Body Weight (kg)	SNa (mEq/L)	Urine Ca/24 h (mg)	Treatment	SNa (mEq/L)	Urine Ca/24 h (mg)	Etiology of SIADH
♂—65—59	128	203	Urea (15 g/day)	133	160	Brain damage
♂—76—60	120	160	Satavaptan (50 mg/day)	139	86	Carbamazepine
♂—74—70	122	360	Urea (30 g/day)	141	34	NSIAD
♂—40—69	125	275	WR	139	192	Carbamazepine
♂—35—76	128	470	WR	139	298	Carbamazepine
♀—83—55	130	155	Urea (15 g/day)	133	77	Idiopathic
♀—27—63	124	434	Urea (30 g/day)	138	102	Brain tumor
♂—18—55	129	240	Urea (30 g/day)	137	80	Brain damage
♀—50—60	120	175	Urea (30 g/day)	136	70	Carbamazepine
52 ± 20 63 ± 6.7	125.1 ± 3.6	275 ± 112		137 ± 2.6 *	122 ± 77 **	

NSIAD: nephrogenic syndrome of inappropriate antidiuresis [19]; * *p* < 0.001; ** *p* < 0.01.

## Data Availability

Not applicable.
